# Improving molecular diagnosis of Chinese patients with Charcot-Marie-Tooth by targeted next-generation sequencing and functional analysis

**DOI:** 10.18632/oncotarget.8377

**Published:** 2016-03-25

**Authors:** Li-Xi Li, Shao-Yun Zhao, Zhi-Jun Liu, Wang Ni, Hong-Fu Li, Bao-Guo Xiao, Zhi-Ying Wu

**Affiliations:** ^1^ Department of Neurology and Institute of Neurology, Huashan Hospital, Institutes of Brain Science and State Key Laboratory of Medical Neurobiology, Shanghai Medical College, Fudan University, Shanghai, 200040, China; ^2^ Department of Neurology and Research Center of Neurology in Second Affiliated Hospital, and The Collaborative Innovation Center for Brain Science, Zhejiang University School of Medicine, Hangzhou 310009, China

**Keywords:** Charcot-Marie-Tooth, molecular diagnosis, targeted next-generation sequencing, genetic variant, functional analysis

## Abstract

Charcot-Marie-Tooth (CMT) disease is the most common hereditary peripheral neuropathy. More than 50 causative genes have been identified. The lack of genotype-phenotype correlations in many CMT patients make it difficult to decide which genes are affected. Recently, targeted next-generation sequencing (NGS) has been introduced as an alternative approach for diagnosis of genetic disorders. Here, we applied targeted NGS in combination with *PMP22* duplication/deletion analysis to screen causative genes in 22 Chinese CMT families. The novel variants detected by targeted NGS were then further studied in cultured cells. Of the 22 unrelated patients, 8 had *PMP22* duplication. The targeted NGS revealed 10 possible pathogenic variants in 11 patients, including 7 previously reported variants and 3 novel heterozygous variants (GJB1: p.Y157H; MFN2: p.G127S; YARS: p.V293M). Further classification of the novel variants according to American College of Medical Genetics and Genomics (ACMG) standards and guidelines and functional analysis in cultured cells indicated that p.Y157H in GJB1 was pathogenic, p.G127S in MFN2 was likely pathogenic, while p.V293M in YARS was likely benign. Our results suggest the potential for targeted NGS to make a more rapid and precise diagnosis in CMT patients. Moreover, the functional analysis is required when the novel variants are indistinct.

## INTRODUCTION

Charcot-Marie-Tooth (CMT) is the most common inherited neuromuscular disorder with an incidence of 40 individuals in every 100 000 inhabitants [1]. The classical symptoms include slowly progressive distal muscle weakness, muscle atrophy and sensory loss of the lower and then upper limbs. As the motor and sensory peripheral nerves are affected, it is also called hereditary sensory and motor neuropathy. On the basis of electrophysiological results, CMT has been traditionally subdivided into two main groups: demyelinating type (CMT1) and axonal type (CMT2).

Thus far, more than 50 disease-causing genes have been identified to be associated with CMT (http://www.molgen.ua.ac.be/CMTMutations/; http://neuromuscular.wustl.edu/) of which the duplication/deletion of *PMP22* is the most common cause of CMT1 [2]. The traditional strategy for molecular diagnosis of CMT is based on the clinical phenotype, inheritance pattern and electrophysiological examination. However, CMT is a highly heterogeneous disorder [3]. It may be inherited in more than one model of inheritance, and a single gene can result in multiple clinical phenotypes. Moreover, there are still many unknown causative genes of CMT waiting to be discovered [4]. It is impractical for a lab to investigate all the possible genes using conventional Sanger sequencing, which is time-consuming and expensive. Consequently, introducing a more comprehensive approach for molecular diagnosis of CMT is important. Targeted next-generation sequencing (NGS), a high-throughput DNA sequencing technology that performs parallel sequencing the genomic regions of interested [5], makes it possible.

Recently, targeted NGS has been successfully performed in the inherited neurologic diseases diagnosis [6–9]. It is an efficient and cost-effective tool for achieving a genetic diagnosis for inherited peripheral neuropathies [10–12]. However, up till now, there is still no literature to evaluate the efficiency of targeted NGS in Chinese CMT patients. Here, we applied targeted NGS in combination with *PMP22* duplication/deletion analysis in a cohort of 22 Chinese CMT families. The novel sequence variants identified by targeted NGS were classified according to the American College of Medical Genetics and Genomics (ACMG) standards and guidelines [13], and further verified by the functional analysis in cultured cells.

## RESULTS

### *PMP22* duplication/deletion analysis

The multiplex ligation-dependent probe amplification (MLPA) analysis was performed in all patients before the examination of targeted NGS. It showed that 8 out of 22 unrelated CMT patients had *PMP22* duplication (Supplemental Table 1). The rest of the CMT patients were further screened for mutations with targeted NGS.

### Identification of variants by targeted NGS and sanger sequencing

Targeted NGS was performed in the remaining 14 patients. Our gene panel included 44 genes (Table 1). The coverage of the fraction of target base can be found in Table 2. Over 99% of target bases had >10x coverage, 97.43% had >30x coverage and 94.24% had >50x coverage. The mean coverage of target bases ranged from 106.86 - 1287.75.

**Table 1:** Target genes included in the panel

Gene	Locus	Ref sequence	MIM	Exons	Gene	Locus	Ref sequence	MIM	Exons
*AARS*	16q22	NM_001605.2	601065	21	*KIF1B*	1p36.2	NM_015074.3	605995	47
*ATL1*	14q22.1	NM_015915.4	606439	14	*KIF5A*	12q13.13	NM_004984.2	602821	29
*ATL3*	11q13.1	NM_015459.4	609369	13	*LITAF*	16p13.13	NM_004862.3	603795	4
*BSCL2*	11q13	NM_032667.6	606158	11	*LRSAM1*	9q33.3	NM_138361.5	610933	25
*DCTN1*	2p13	NM_004082.4	601143	32	*MARS*	12q13.3	NM_004990.3	156560	21
*DHTKD1*	10p14	NM_018706.6	614984	17	*MFN2*	1p36.22	NM_014874.3	608507	19
*DNM2*	19p13.2	NM_001005361.2	602378	21	*MPZ*	1q23.3	NM_000530.7	159440	6
*DNMT1*	19p13.2	NM_001130823.2	126375	41	*NAGLU*	17q21	NM_000263.3	609701	6
*DYNC1H1*	14q32	NM_001376.4	600112	78	*NEFL*	8p21	NM_006158.4	162280	4
*EGR2*	10q21.1	NM_000399.3	129010	2	*PDK3*	Xp22.11	NM_001142386.2	300906	12
*GARS*	7p15	NM_002047.3	600287	17	*PMP22*	17p12	NM_000304.3	601097	5
*GDAP1*	8q21.11	NM_018972.2	606598	6	*RAB7*	3q21.3	NM_004637.5	602298	6
*GJB1*	Xq13.1	NM_001097642.2	304040	2	*SCN11A*	3p22.2	NM_001287223.1	604385	26
*GNB4*	3q26.33	NM_021629.3	610863	10	*SEPT9*	17q25	NM_001113493.1	604061	11
*HARS*	5q31.3	NM_001289094.1	142810	13	*SLC5A7*	2q12	NM_001305005.2	608761	9
*HOXD10*	2q31.1	NM_002148.3	142984	2	*SOX10*	22q13.1	NM_006941.3	602229	4
*HSPB1*	7q11.23	NM_001540.3	602195	3	*SPTLC1*	9q22.2	NM_001281303.1	605712	15
*HSPB3*	5q11.2	NM_006308.2	604624	1	*SPTLC2*	14q24.3	NM_004863.3	605713	12
*HSPB8*	12q24.23	NM_014365.2	608014	3	*TFG*	3q12.2	NM_001195478.1	602498	8
*IFRD1*	7q31.1	NM_001550.3	603502	12	*TRPV4*	12q24.1	NM_021625.4	605427	16
*INF2*	14q32.33	NM_001031714.3	610982	22	*VCP*	9p13.3	NM_007126.3	601023	17
*KARS*	16q23.1	NM_001130089.1	601421	15	*YARS*	1p35.1	NM_003680.3	608323	13

Abbreviations: MIM = Mendelian Inheritance in Man.

**Table 2:** The coverage of the fraction of target bases

	% fraction of target bases					Mean coverage of target bases
	coverage > 10x	coverage > 20x	coverage > 30x	coverage > 40x	coverage > 50x	
Mean	99.36	98.53	97.43	96.08	94.24	649.39
Range	97.60 - 99.91	93.83 - 99.84	88.90 - 99.75	82.97 - 99.66	76.13 - 99.38	106.86 - 1287.75

On average, 105 variants were identified in each CMT patient. After being filtered and verified by Sanger sequencing, 10 possible pathogenic variants, including 7 previously reported ones (GJB1: p.V91M, p.R164Q, p.R164W, p.R183H; MFN2: p.R280H; HSPB8: p.K141N; BSCL2: p.S90L) and 3 novel ones (GJB1: p.Y157H; MFN2: p.G127S; YARS: p.V293M), were identified in 11 cases (2 cases carrying the p.R164W in GJB1, Table 3). No possible pathogenic variant was found in the remaining 3 patients (Supplemental Table 1).

**Table 3:** Clinical manifestations of 11 individuals with genetic variants detected by targeted NGS

Characteristics	Patients										
	case 2	case 5	case 6	case 7	case 9	case 13	case 15	case 17	case 18	case 19	case 20
Age at study, (years)	17	43	16	43	23	39	56	15	26	32	20
Sex	Female	Male	Male	Male	Female	Male	Male	Male	Male	Female	Male
Age at onset, (years)	6	30	10	27	21	36	41	10	11	29	10
Inheritance	D	D	D	D	D	D	D	D	D	D	D
Variants	MFN2	MFN2	GJB1	YARS	GJB1	HSPB8	GJB1	GJB1	GJB1	GJB1	BSCL2
Nucleotide	379G>A	839G>A	491G>A	877G>A	469T>C	423G>C	490C>T	548G>A	490C>T	271G>A	269C>T
Amino acid	G127S	R280H	R164Q	V293M	Y157H	K141N	R164W	R183H	R164W	V91M	S90L
Novel or known	Novel	Known	Known	Novel	Novel	Known	Known	Known	Known	Known	Known
Muscle weakness UL	Yes	Not	Not	Not	Not	Not	Yes	Yes	Yes	Yes	Not
Muscle weakness LL	Yes	Yes	Yes	Yes	Yes	Yes	Yes	Yes	Yes	Yes	Yes
Muscle atrophy UL	Yes	Not	Not	Not	Not	Not	Not	Yes	Yes	Yes	Not
Muscle atrophy LL	Yes	Yes	Yes	Yes	Not	Yes	Not	Yes	Yes	Yes	Not
Ankle DTRs	Reduced	Absent	Absent	Absent	Absent	Absent	Absent	Absent	Absent	Absent	Reduced
Median nerve, R/L											
cMAP, mV	1.1/NA	NA/NA	0.2/1.7	0.6/1.4	5.8/3.7	6.7/NA	0.9/NA	12.1/8.1	NA/NA	1.2/2.7	1.5/NA
MNCV, m/s	37.8/NA	NA/NA	32.0/34.4	27.8/31.9	46.0/42.3	56.0/NA	35.3/NA	40.8/38.8	NA/NA	40.7/40.4	61.5/NA
Peroneal nerve, R/L											
cMAP, mV	NP/NA	1.2/5.3	NP/NA	NP/NA	0.1/NA	1.4/NA	NP/NA	1.9/2.2	NA/NA	NA/NA	3.0/2.2
MNCV, m/s	NP/NA	35.1/37.8	NP/NA	NP/NA	34.9/NA	40.8/NA	NP/NA	40.7/40.9	NA/NA	NP/NA	36.5/37.4
Median nerve, R/L											
SNAP, uv	14/NA	NA/NA	3.8/0.7	NP/NP	1.6/3.6	25/NA	5.4/NA	0.84/1.3	NA/NA	9.5/8.7	13.2/NA
SNCV, m/s	50/NA	NA/NA	39.4/34.6	NP/NP	44.4/43.1	56.5/NA	37.8/NA	36.6/40.7	NA/NA	44.8/44.8	50/NA
Sural nerve, R/L											
SNAP, uv	1.4/NP	NA/NA	NP/NP	NP/NP	NP/NP	NA/19	3.7/4.0	3.9/5.0	NA/NA	4.8/NA	23.1/16.6
SNCV, m/s	26.3/NP	NA/NA	NP/NP	NP/NP	NP/NP	NA/46.8	36.2/38.3	36.8/36.5	NA/NA	36.7/NA	41/40
Laboratory test											
CK, U/L	NA	NA	290	NA	212	744	NA	NA	NA	200	NA
LDH, U/L	NA	NA	227	NA	242	255	NA	NA	NA	172	NA

Abbreviations: D = dominant; NA = not available; NP = not potential; MNCV = motor nerve conduction velocity; cMAP = compound muscle action potential; SNAP = sensory nerve action potential; SNCV = sensory nerve conduction velocity; R/L = right/left.

Normal values: median MNCV ≥ 49 m/s; median cMAP ≥ 4.5 mv; peroneal MNCV ≥ 40 m/s; peroneal cMAP ≥ 2.5 mv; median SNCV ≥ 50 m/s; median SMAP ≥ 5.0 uv; sural SNCV ≥ 40 m/s; sural SMAP ≥ 5.0 uv.

Reference range: CK < 174 U/L; LDH < 211 U/L.

### Classification of novel variants and functional analysis

The three novel variants were absent in 1000Genomes and dbSNP database and were not present in 500 control subjects. The SIFT and PolyPhen-2 software programs were used to predict the functional disruption of proteins due to amino acid change.

**Figure 1:**
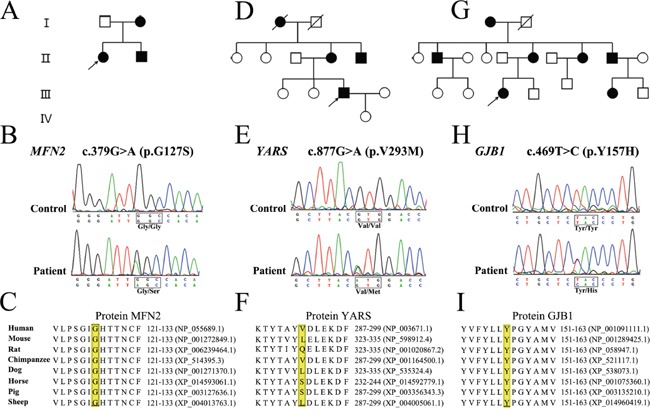
Pedigree, sequencing chromatograms and conservation analysis of novel variants detected by targeted NGS **A-C.** Pedigree of the proband with novel variant in *MFN2*, *YARS* and *GJB1*, respectively. **D-F.** Electropherograms showing the sequence variation in *MFN2*, *YARS* and *GJB1*, respectively. **G-I.** Conservation analysis. YARS, MFN2 and GJB1 protein sequence alignments from multiple, evolutionarily diverse species were depicted.

The novel variant c.379G>A (p.G127S) in *MFN2* affects the same amino acid as two previously reported mutations (p.G127D and p.G127V) [14, 15]. This variant was further identified in the proband's affected mother and younger brother who presented with a similar phenotype, while her unaffected father did not carry the same variant (Figure 1A and 1B). The amino acid change was predicted to be deleterious by SIFT (score: 0) and PolyPhen-2 (score: 1.0). Additionally, the variant site was conserved in different animal species (Figure 1C). According to ACMG standards and guidelines, this variant was classified as a likely pathogenic variant.

The novel variant c.877G>A (p.V293M) in *YARS* (Figure 1D and 1E) was predicted to be deleterious by the SIFT (score: 0.02), while benign in PolyPhen-2 (score: 0.049). The variant site was not conserved in different animal species (Figure 1F). The segregation analysis was not available as the blood samples were unobtainable from other family members. In contrast to all previously identified *YARS* mutations [16], this novel variant was located at the anticodon recognition region of YARS protein.

The novel variant c.469T>C (p.Y157H) in *GJB1* (Figure 1G and 1H) affected the same amino acid as the previously reported mutation (p.Y157C) [17]. The variant site was conserved in different animal species (Figure 1I) and was predicted to be deleterious by SIFT (score: 0) and PolyPhen-2 (score: 1.0). However, segregation analysis was not performed, as no other family members were available for further examination.

**Figure 2:**
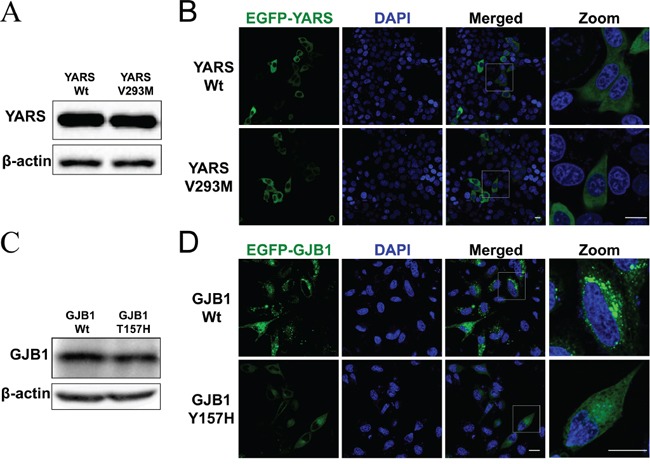
Functional analysis in cultured cells **A.** Western blot analysis of protein extracts obtained from HEK293 cells transfected with pFlag-YARS-Wt or pFlag-YARS-V293M vectors. The anti-Flag antibody was used to detect the interested band. **B.** Confocal images of HeLa cells transiently transfected with pEGFP-YARS-Wt or pEGFP-YARS-V293M vectors. Transfected cells were examined for green fluorescence 48 hours after transfection. Cell nuclei were stained with 4′-6-diamidino-2-phenylindole (DAPI; blue). Scale bar, 20 μm. **C.** Western blot analysis of GJB1 expression in HEK293 cells transfected with pEGFP-GJB1-Wt or pEGFP-GJB1-Y157H vector. The anti-GFP antibody was used to detected recombinant proteins. **D.** Confocal images of HeLa cells transiently transfected with pEGFP-GJB1-Wt or pEGFP-GJB1-Y157H constructs. Cells expressing wild type GJB1 displayed granules throughout the cytosol, whereas cells expressing the mutant GJB1 had diffuse intracellular staining. Scale bar, 20 μm.

To determine the consequence of amino acid change in YARS or GJB1, functional analysis in cultured cells was further performed. The data revealed that HEK293 cells transfected with wild-type or mutant *YARS* had the comparable mRNA (Supplementary Figure 1) and protein level (Figure 2A). Furthermore, the p.V293M variant did not change the protein's distribution in HeLa cells (Figure 2B), HEK293 cells or SH-SY5Y neuroblastoma cell lines (data not shown). These studies suggested that the novel variant p.V293M in *YARS* was likely benign. With regard to GJB1, we found that the p.Y157H change did not affect the GJB1 mRNA (Supplementary Figure 1) or protein level (Figure 2C). The fluorescence study revealed that HeLa cells transfected with EGFP-GJB1-Wt formed intracellular granules, whereas cells expressed EGFP-GJB1-Y157H had diffuse intracellular staining (Figure 2D). These data indicated that p.Y157H variant affected intracellular distribution of GJB1, suggesting the pathogenicity of this novel variant.

### Clinical features of CMT patients

The clinical features of recruited CMT patients were summarized in Supplemental Table 1. Among these patients, 17 were male and 5 were female, and ages ranged from 12 to 66. All these cases displayed a progressive phenotype. In the majority of CMT patients, the symptoms at disease onset were muscle weakness in the lower limbs. Motor deficits were more marked distally and more sever in the lower limbs.

The patient (case 2) carrying the likely pathogenic variant p.G127S in MFN2 was a 17-year-old female (Table 3). The symptoms and signs were began at age of 6 with gait disturbance. At the age of 15, she underwent orthopedic surgery. The clinical examinations revealed reduced deep tendon reflex, muscle weakness in the distal limbs and muscle atrophy in both hands. Conduction velocity of the median nerve was reduced, with a moderate decrease in distal amplitudes.

The patient (case 7) carrying the likely benign variant p.V293M in YARS was a 43-year-old male (Table 3). He displayed foot drop, distal muscle weakness and atrophy in the lower limbs for about 18 years. He did not feel numbness in the limbs. Neurological examinations revealed bilateral foot drop with pes cavus, muscle weakness and atrophy in the lower limbs, and areflexia in all limbs. He had horizontal nystagmus. The motor nerve conduction velocity and muscle compound action potential were markedly reduced in all limbs.

The patient (case 9) carrying the p.Y157H pathogenic variant in GJB1 was a 23-year-old female from a large family with several affected members (Table 3). Onset of the disease was at age 21 years, with distal weakness in legs and steppage gait. Neurological examinations revealed pes cavus, reduced muscle strength in the distal lower limbs and areflexia in all limbs. Electrophysiological studies showed reduced nerve conduction and compound muscle action potential in the peroneal nerve.

## DISCUSSION

There are a number of different NGS technologies, including whole genome sequencing (WGS), whole exome sequencing (WES) and targeted NGS. All these technologies have already been successfully performed in CMT patients [18–20]. While it seems that WGS and WES are more attractive to identify underlying genetic causes than targeted NGS, one of the drawbacks of WES and WGS is that they generate a huge amount of unnecessary data. It is difficult and time-consuming to interpret those variants, especially when only one patient is available for sequencing. For targeted NGS, the panel covering a determined set of candidate genes can be designed to screen causative mutations. It offers some advantages owing to cost savings and the speed of data interpretation [5, 21]. Therefore, in the current study, we introduced targeted NGS to detect the causative genes in Chinese CMT patients. Since targeted NGS was relatively less effective to detect the copy number variants [12, 22], the *PMP22* duplication/deletion was firstly analyzed.

Thus far, targeted NGS approach has been applied to CMT patients [11, 12, 18]. Just like these studies, our results demonstrated that targeted NGS is an effective method to make a molecular diagnosis in CMT patients. However, to compare with those studies, there are some differences in our study. Firstly, apart from the *PMP22* duplication/deletion analysis, the other common causative genes, such as *GJB1* and *MFN2*, had not been detected before the examination of targeted NGS. The published studies have revealed that over 90% of the CMT patients carried mutations in *PMP22*, *MPZ*, *GJB1* and *MFN2* [4, 23]. In our study, 8 cases had mutations in *GJB1* and *MFN2*. Secondly, a specific dominant gene panel was designed in our study. Therefore, we recruited CMT families with dominant inheritance pattern. For targeted NGS, the most significant difficulty is how to analyze and interpret the possible pathogenic variants. To overcome this obstacle, a more logical approach is to design a smaller panel that covers major subtypes. The disease subtype-specific NGS panel costs less than the complete CMT gene panel, and the number of irrelevant variants is reduced [5]. In our study, variants identified in the dominant CMT gene panel were fewer than the complete CMT gene panel [11]. Lastly, we made a molecular diagnosis in 10 patients using targeted NGS (case 7 carrying a likely benign variant). The diagnostic success rate is higher than the previously reported in the literatures [10–12].

After being interpreted, 10 possible pathogenic variants were found in our study. The clinical characteristics of these CMT patients with known variants are comparable to the reported data [15, 24–28]. In case 2, a likely pathogenic novel variant in *MFN2* was found. MFN2 is localized in the outer mitochondrial membrane which contains two hydrophobic heptad repeat domains and a GTPase domain. The novel variant was located at the GTPase domain. An intact GTPase domain is indispensable for the function of mitochondrial fusion [29]. In case 9, a novel pathogenic variant in *GJB1* was identified. Functional analysis revealed that wild-type GJB1 formed puncta staining in the mammalian cells, while this phenomenon was disappeared when the cells expressed GJB1 mutant (p.Y157H). The result was consistent with the previously reported GJB1 mutants (p.M34K, p.N205I and p.Y211X). Many GJB1 mutants showed abnormal trafficking when expressed in mammalian cells [30].

In case 7, the novel variant in *YARS* was detected. *YARS*, encoding tyrosyl-tRNA synthetase (TyrRS), is an aminoacyl-tRNA synthetase involved in dominant-intermediate CMT [16, 31]. TyrRS contains three functional domains: an N-terminal catalytic domain, a central anticodon recognition domain and a C-terminal EMAP II-like domain. So far, all previously identified pathogenic TyrRS mutations, including p.G41R, p.E196K and p.153-156delVKQV, were all located at the catalytic domain of the protein. These mutations affect the protein's localization in sprouting neurites of neuroblastoma cells [16]. A missense p.K265N variant located at the anticodon recognition domain of TyrRS has been reported. However, it was demonstrated to be a benign polymorphism [32]. The novel variant we found was also located at the anticodon recognition domain of TyrRS. Further functional analysis revealed that this variant did not change the protein's expression or intracellular localization. Therefore, this novel variant in *YARS* was classified as likely benign.

Even after targeted NGS, 4 cases (including case 7) still did not have a molecular diagnosis. There may be several reasons for this. First of all, as the filtering method was too strict, it's possible that some truly pathogenic variants were recognized as benign. Secondly, in addition to the copy number variations (CNVs) of *PMP22*, we did not detect any further CNVs in other causative genes. Although CNVs outside of *PMP22* locus are rare, it is still important to test them in CMT cases negative for the *PMP22* duplication [33–35]. Finally, there are still many other CMT causative-genes that remain to be identified. It has been estimated that 50% of CMT patients may carry mutations in unknown disease genes [4].

In summary, we have combined targeted NGS and *PMP22* duplication/deletion analysis as a diagnostic strategy for CMT patients. Besides 7 previously reported variants, we found 3 novel variants after targeted NGS analysis. Further classification of sequence variants according to ACMG standards and guidelines and functional analysis in cultured cells indicated that p.Y157H in GJB1 was pathogenic, p.G127S in MFN2 was likely pathogenic, while p.V293M in YARS was likely benign. Although the number of cases was small, our results still revealed an increase in diagnostic success rate.

## MATERIALS AND METHODS

### Patients

Patients were recruited from the Department of Neurology, Huashan Hospital, and Second Affiliated Hospital of Zhejiang University School of Medicine from December 2007 to June 2015. Patients were considered to suffer from CMT if they had a sensorimotor peripheral neuropathy (according to their medical history, neurological examination and neurophysiological testing) and a family history of similar characteristics. The clinical diagnostic strategy was performed as described in the previously reported literature [23]. In total, 22 Chinese CMT families with dominant inheritance pattern were enrolled. Clinical evaluations were carried out by at least two senior neurologists. Routine blood biochemical tests and electrophysiological tests were performed. Five hundred unrelated aged individuals (≥65 years) without history of CMT were selected as a control group. Written informed consents were obtained from all the participants. This study was approved by the ethics board of Huashan Hospital and Second Affiliated Hospital.

### *PMP22* duplication/deletion analysis

All the CMT patients were tested for the *PMP22* duplication/deletion using MLPA as reported previously [36]. MLPA was performed using the MLPA kit (MRC Holland, Netherlands), according to the manufacture's protocol.

### Targeted NGS

Genomic DNA was extracted from peripheral EDTA-treated blood using Blood Genomic Extraction Kit (Qiagen, Germany). A dominant gene panel was designed to cover 44 genes known to be associated with dominant CMT and other inherited peripheral neuropathies (Table 1). Genetic features can be found in the online databases including Neuromuscular Disease Center (http://neuromuscular.wustl.edu/time/hmsn.html) and Inherited Peripheral Neuropathies Mutation Database (http://www.molgen.ua.ac.be/cmtmutations/). All of the exons and the 20 flanking base pairs of the splice junctions surrounding the exons of targeted genes were included. The samples were captured by NimbleGen SeqCap EZ products (Roche, Switzerland). Deep sequencing was further done on an Illumina HiSeq2000 platform (Genergy Biotechnology Co Ltd, Shanghai, China). Each read was aligned to the hg19 reference genome (http://hgdownload.cse.ucsc.edu/) using the Burrows-Wheeler Aligner (BWA, version 0.7.12-r1039) [37]. The variants were detected using Genome Analysis Toolkit (GATK, version 3.1-1-g07a4bf8) following the GATK best practices [38]. All the variants were annotated by the ANNOVAR (Version 2014-11-12). Variants were further filtered, as described in our previously publication [39]. Two software programs, SIFT (http://sift.jcvi.org/) and PolyPhen-2 (http://genetics.bwh.harvard.edu/pph2/), were used to predict the possible protein functional change caused by the variant.

### Sanger sequencing

Sanger sequencing was used to validate all the potential variants using standard protocols. PCR was performed to amplify the fragments covering the variant sites. The PCR products were purified and further directly sequenced on ABI 3730 DNA Sequencer. The sequencing results were aligned to human reference genome published in Ensembl (http://www.ensembl.org/).

### Functional studies in cultured cells

The cDNA encoding human *GJB1* was cloned into HindIII/KpnI site of pEGFP-C2 vector. The cDNA encoding human *YARS* was cloned into HindIII/KpnI site of pFLAG-CMV-4 vector and pEGFP-C2 vector. All mutant constructs of *GJB1* and *YARS* were created by PCR mutagenesis and verified by Sanger sequencing.

HEK293 and HeLa cells were cultured in DMEM supplemented 10% fetal bovine serum in a 5% CO_2_ incubator. To explore whether the novel variant affected mRNA and protein expression, HEK293 cells were transfected with wild-type, mutant expressing or empty vectors. Transient transfection was performed using the Lipofectamine 2000 according to the manufacture's protocol (Invitrogen, USA). Forty-eight hours after transfection, cells were lysed and harvested. The mRNA expression level of *YARS* and *GJB1* was detected using RT-PCR analysis. The following primers were used: *GJB1* Forward: GCGTGAACCGGCATTCTACT; *GJB1* Reverse: TTGGTCATAGCAAACGCTGTT; *YARS* Forward: CTGCACCTTATCACCCGGAAC; *YARS* Reverse: TCCGCAAACAGAATTGTTACCT; *GAPDH* Forward: ACTCCACGACGTACTCAG; *GAPDH* Reverse: CATGTTCCAATATGATTCCACC. The protein samples were resolved by SDS-PAGE, transferred to nitrocellulose membrane and blotted with the desired antibodies. The antibodies against Flag (1:5 000; Abmart, China), GFP (1:5 000; Santa Cruz, USA) and β-actin (1:5 000; Sigma-Aldrich, USA) were used.

To further define the intracellular distribution of wild-type and mutant protein in mammalian cell lines, HeLa cells transiently over-expressing EGFP-YARS-Wt, EGFP-YARS-V293M, EGFP-GJB1-Wt, EGFP-GJB1-Y157H or the empty vector were directly observed under a confocal microscope (Leica, Germany).

## SUPPLEMENTARY FIGURE AND TABLE


