# Supplementary material for: Improving molecular diagnosis of Chinese patients with Charcot-Marie-Tooth by targeted next-generation sequencing and functional analysis
Source: Oncotarget. 2016 Mar 25;7(19):27655–64. doi: 10.18632/oncotarget.8377 (PMC5053678; doi:10.18632/oncotarget.8377)
Supplement: Supplementary file 1 [file oncotarget-07-27655-s001.pdf]

## SUPPLEMENTARY FIGURE AND TABLE

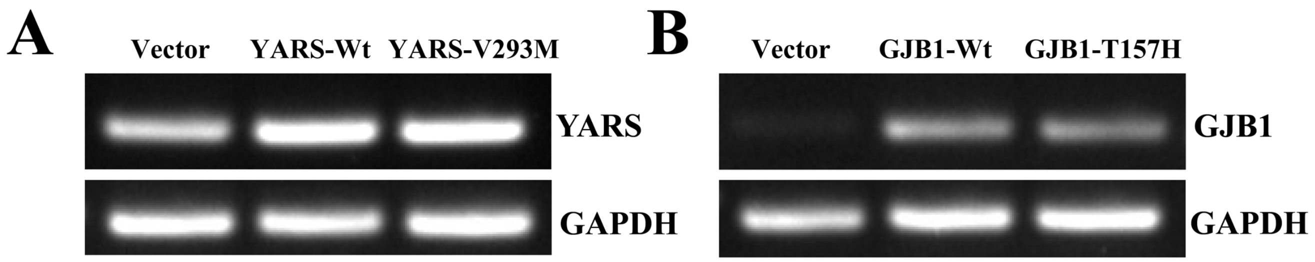


**Supplementary Figure S1: The missense mutations did not affect mRNA expression level of GJB1 A. or YARS B. in HEK293 cells.**

**Supplementary Table S1: Clinical characteristics of the enrolled CMT patients**

| Case/ Sex | Family history | Age at onset/ examination (years) | Symptoms at disease onset | Gene | Nucleotide | Amino acid change | Variants known or novel | SIFT/ Polyphen-2 score |
|---|---|---|---|---|---|---|---|---|
| 1/M | Yes | 12/16 | Gait disturbance | - | - | - | - | - |
| 2/F | Yes | 6/17 | Gait disturbance | MFN2 (NM_014874.3) | c.379G>A | p.G127S | Novel | 0 / 1.0 |
| 3/M | Yes | 11/12 | Distal muscle weakness in the low limbs | PMP22 | Duplication | - | - | - |
| 4/M | Yes | 44/52 | Distal muscle weakness in the low limbs | PMP22 | Duplication | - | - | - |
| 5/M | Yes | 30/43 | Distal muscle weakness in the low limbs | MFN2 (NM_014874.3) | c.839G>A | p.R280H | Known | - |
| 6/M | Yes | 10/16 | Distal muscle weakness in the low limbs | GJB1 (NM_001097642.2) | c.491G>A | p.R164Q | Known | - |
| 7/M | Yes | 27/43 | Foot drop and weakness in the low limbs | YARS (NM_003680.3) | c.877G>A | p.V293M | Novel | 0.02 / 0.049 |
| 8/M | Yes | 22/32 | Muscle weakness in all limbs | PMP22 | Duplication | - | - | - |
| 9/F | Yes | 21/23 | Weakness in the low limbs | GJB1 (NM_001097642.2) | c.469T>C | p.Y157H | Novel | 0 / 1.0 |
| 10/M | Yes | 23/24 | Difficulty in dorsiflexion of the left ankle | - | - | - | - | - |
| 11/M | Yes | 5/35 | Distal muscle weakness in the low limbs | PMP22 | Duplication | - | - | - |
| 12/M | Yes | childhood/14 | Delayed motor milestones | - | - | - | - | - |
| 13/M | Yes | 36/39 | Distal muscle weakness and atrophy in the low limbs | HSPB8 (NM_014365.2) | c.423G>C | p.K141N | Known | - |
| 14/F | Yes | childhood/25 | Difficulty in running | PMP22 | Duplication | - | - | - |
| 15/M | Yes | 41/56 | Difficulty in walking and dorsiflexion | GJB1 (NM_001097642.2) | c.490C>T | p.R164W | Known | - |
| 16/F | Yes | 46/66 | Difficulty in hand manipulation | PMP22 | Duplication | - | - | - |
| 17/M | Yes | 10/15 | Difficulty in running and twisting of the ankle | GJB1 (NM_001097642.2) | c.548G>A | p.R183H | Known | - |

(*Continued*)

| Case/ Sex | Family history | Age at onset/ examination (years) | Symptoms at disease onset | Gene | Nucleotide | Amino acid change | Variants known or novel | SIFT/ Polyphen-2 score |
|---|---|---|---|---|---|---|---|---|
| 18/M | Yes | 11/26 | Distal muscle weakness in the low limbs | GJB1 (NM_001097642.2) | c.490C>T | p.R164W | Known | - |
| 19/F | Yes | 29/32 | Muscle weakness in the right leg | GJB1 (NM_001097642.2) | c.271G>A | p.V91M | Known | - |
| 20/M | Yes | 10/20 | Muscle weakness in the low limbs | BSCL2 (NM_032667.6) | c.269C>T | p.S90L | Known | - |
| 21/M | Yes | 38/44 | Distal muscle atrophy in the upper limbs | PMP22 | Duplication | - | - | - |
| 22/M | Yes | 21/51 | Distal muscle weakness and atrophy in the low limbs | PMP22 | Duplication | - | - | - |

**Abbreviation:** M=male; F=female.
